# Maternal antibiotic treatment during pregnancy attenuates the transport and absorption of maternal antibody IgG through TLR4 and TLR2 receptor

**DOI:** 10.3389/fmicb.2023.1109273

**Published:** 2023-02-17

**Authors:** Yanan Ding, Xiaofeng Yao, Haihan Zhang, Xi He, Zehe Song

**Affiliations:** College of Animal Science and Technology, Hunan Agricultural University, Changsha, China

**Keywords:** gut microbiota, antibiotic, toll like receptor, IgG transport, IgG absorption

## Abstract

Maternal antibody IgG, the main antibody in colostrum, plays an important role in neonates protection. Commensal microbiota is closely related to host antibody repertoire. However, there are few reports on how maternal gut microbiota affects maternal antibody IgG transfer. In the present study, we investigated the effects of altering the gut microbiota (treated with antibiotics during pregnancy) on maternal IgG transportation and offspring absorption and explored its underlying mechanisms. Results showed that antibiotic treatment during pregnancy significantly decreased maternal cecal microbial richness (Chao1 and Obesrved species) and diversity (Shannon and Simpson). Plasma metabolome enriched significant changes in the process of bile acid secretion pathway, and the concentration of deoxycholic acid, a secondary metabolite of microorganisms was lowered. Flow cytometry analysis indicated that antibiotic treatment promoted the number of B cells and abated the number of T, DC and M1 cells in intestinal lamina propria of dams. Surprisingly, the serum IgG level in antibiotic treated dams was significantly increased, while IgG contents in colostrum was decreased. Moreover, pregnancy antibiotic treatment in dams was reduced the expression of FcRn, TLR4 and TLR2 in breast of dams and in duodenum and jejunum of neonates. Furthermore, TLR4^−/−^ and TLR2^−/−^ knock-out mice showed a lower FcRn expression in breast of dams and in duodenum and jejunum of neonates. These findings suggest that maternal intestine bacteria may affect the maternal IgG transfer through regulating the breast TLR4 and TLR2 of dams.

## Introduction

Neonates are highly susceptible to microbial infections, not only as their immature immune system which is inability to produce adaptive immune effectors such as antibodies (Basha et al., 2014; McMichael et al., 2015), but also their immature intestine microbiota which hardly to against pathogens (Kamada et al., 2013). Passive immunity to various pathogenic bacterial and viral infections (such as group *B Streptococcus*, *Haemophilus influenzae* and influenza viruses) can be transferred to neonates through maternal antigen-specific IgG antibodies induced by maternal colonization or vaccination (Madoff et al., 1992; Zaman et al., 2008). Neonates acquire maternal antibodies through the placenta and breast milk. Human and rodent milk contains substantial amounts of IgA and IgG, and the content of IgG in colostrum is higher than IgA (Goldsmith et al., 1983; Fouda et al., 2011). Thus, breast-feeding provided IgG could mediate protection at the mucosa or submucosal surface to prevent invasive pathogenic bacterial species.

The commensal microbiota was reported to shape the antibody repertoire (Macpherson et al., 2017; Chen et al., 2018). Study found that murein lipoprotein (MLP), an outer membrane protein conserved in gram-negative symbiotic bacteria and pathogens, drives protective IgG in mice (Zeng et al., 2016). As a commensal microbiota species, lactose-non-fermenting gram-negative *Enterobacteriaceae speciesa Pantoea* can elicit cross-reactive antibodies IgG against ETEC (Zheng et al., 2020). Moreover, microbes can affect the absorption of IgG mediated by FcRn, the only receptor that specifically transports IgG in neonates (Zheng et al., 2020). Studies found that FcRn is also expressed in the breast of quite a few animals, such as pigs, rodents, ruminants and humans, which can directly transport IgG by combining with Fc fragments of IgG, and participate in maintaining the dynamic balance of IgG (Lu et al., 2007; Bournazos and Ravetch, 2017). Nevertheless, there are few reports on how microbiota affect the transport of IgG from maternal to colostrum and the absorption of IgG in the intestine of neonates.

Therefore, this experiment was conducted to investigate how the maternal intestine bacteria affect the production of maternal IgG and its transfer to neonates. In the current study, we discovered that antibiotic treatment in pregnancy down-regulated FcRn expression *via* TLR4 and TLR2 receptors, hence reducing the transport of IgG from maternal serum to colostrum, and of IgG from colostrum to serum.

## Materials and methods

### Materials

Cefoperazone sodium samples (purity >98%) were purchased from Shanghai Yuanye Biotechnology Co., Ltd. (Shanghai, China).

### Animal and experimental design

After approval by the Committee on the Ethics of Animal Experiments of Hunan Agriculture University, female 9-week-old C57BL/6 mice, purchased from the Hunan SJA Laboratory Animal Co., Ltd. (Hunan, China), were mated by housing one female and an adult male per cage for 1 night and the adult male mice were removed in the next morning. Ten days later, the size of the mouse belly was observed to determine whether it was successful in pregnancy. Then dams were randomly divided into 2 groups (12 dams per group). The control group received regular feeding and drinking water, and the antibiotic group received 0.5 g/L cefoperazone sodium in drinking water on the 15th day of pregnancy until the day of delivery. The trial ended at the 3rd day of lactation. The C57BL/6J TLR4^−/−^, and TLR2^−/−^ mice were provided by the Shanghai Model Organisms Center, Inc. (Shanghai, China). After breeding, the experiment shall be conducted according to the above breeding procedure.

### Sample preparations

Blood samples were collected from the orbital plexus and the mice were sacrificed. Colostrum sample is taken from stomach of pups. Breast tissue of the dams and the intestinal tissue of the pups was removed, rinsed with a physiological saline solution, and stored at −80°C until analyzed. Meanwhile, cecal contents were collected in eppendorf tubes and immediately stored at −80°C for subsequent analysis. Intestinal tract of the dams was removed and then used for pretreatment of flow cytometry.

### Real-time quantitative PCR

Total RNA was extracted from the breast of dams and duodenum and jejunum of offspring using EasyPureTM RNA kit (Beijing Transgene Biotech Ltd., Beijing, China). Use reverse transcription kit (TransGen Biotech Co., Ltd., Beijing, China) to obtain cDNA samples. Real-time PCR for analysis of the gene expression was performed using SYBR Green (Thermo Fisher Scientific, MA, United States) on an ABI 6 flex real-time PCR instrument (Thermo Fisher Scientific, MA, United States). The PCR sequence of each primer is shown in Supplementary Table S1.

### 16s rRNA gene sequencing

Total bacterial genomic DNA samples were extracted using a Fast DNA SPIN extraction kit (MP Biomedicals, Santa Ana, CA, United States), following the manufacturer’s instructions. The quantity and quality of the extracted DNA was measured using a NanoDrop ND-1000 spectrophotometer (Thermo Fisher Scientific, Waltham, MA, United States) and agarose gel electrophoresis, respectively. PCR amplification of the nearly full-length bacterial 16S rRNA genes was performed using the forward primer (5′-AGAGTTTGATCMTGGCTCAG-3′) and reversed primer (5′-ACCTTGTTACGACTT-3′). Sequence data analyses were mainly performed using QIIME (v1.8.0) and R packages (v 3.2.0), including the quality control of raw data, taxonomic annotation according to NCBI database. Use the ASV/OTU table that is not flattened, call the “qiime diversity alpha rarefaction” command, set the parameter “-- p-steps 10 -- p-min-depth 10 -- p-iterations 10,” and calculate the selected alpha diversity index. The average score at the maximum leveling depth is selected as the alpha diversity index. Use the flattened ASV/OTU table to call the “qiime diversity core metric physical” or “qiime diversity core metrics” command to perform PCoA analysis, and use the R script to perform PCoA analysis and output the PCoA coordinates of the sample points, and draw them into a two-dimensional scatter plot. Use QIIME2 to call the “qiime taxa barblot” command to make a visual diagram of the composite distribution of samples at the level of phylum and genus classification.

### Plasma nontargeted metabonomics

Fourteen maternal rat plasma samples were selected for plasma non-targeted metabonomic analysis. Samples were tested by quality inspection, mass spectrometry and bioinformatics analysis, and finally presented in the form of data. The raw data were firstly transformed to mzXML format by MSConvert in ProteoWizard software package (v3.0.8789; Smith et al., 2006) and processed using XCMS (Navarro-Reig et al., 2015) for feature detection, retention time correction and alignment. The metabolites were identified by accuracy mass (<30 ppm) and MS/MS data which were matched with HMDB (Wishart et al., 2007),^1^ massbank (Horai et al., 2010),^2^ LipidMaps (Sud et al., 2007),^3^ mzclound[10],^4^ and KEGG (Ogata et al., 1999).^5^ The robust LOESS signal correction (QC-RLSC; Gagnebin et al., 2017) was applied for data normalization to correct for any systematic bias. After normalization, only ion peaks with relative standard deviations (RSDs) less than 30% in QC were kept ensuring proper metabolite identification. The Ropls (Thévenot et al., 2015) software was used for all multivariate data analyses and modeling. Data were mean-centered using scaling. Models were built on principal component analysis (PCA). Differential metabolites were subjected to pathway analysis by MetaboAnalyst (Xia and Wishart, 2011), which combines results from powerful pathway enrichment analysis with the pathway topology analysis.

### Flow cytometry

Use the Lamina Propria Dissociation Kit (Miltenyi biotech, North Rhine Westphalia, Germany) to dissociate the lamina propria cells of the dams intestine and prepare them into a single cell suspension for flow cytometry. The surface markers of intestinal lamina propria lymphocytes were stained as follows. For extracellular markers, single cells were stained at 2 × 10^6^ cells per well in a 96-well V bottomed plate. T and B cells were stained with anti-CD3 BV421, anti-CD23 FITC, anti-CD21 BB700, anti-B220-PE, anti-CD19 APC, anti-CD45 APC-CY7 (BD, NJ, United States). DC and macrophage cells were stained with anti-F4/80 BV421, anti-MHCII PERCP-CY5.5, anti-CD11c APC (BD, NJ, United States). Cells were acquired on an LSRII (Becton Dickinson) and analyzed by FlowJo (Tree Star, Ashland).

### Antibody IgG and LPS ELISA

Colostrum samples were collected from pups’ stomach 3 days postpartum. Relative levels of IgG level were determined by antibody ELISA, purchased from Jiangsu Enzyme Tag Biotechnology Co., LTD (Jiangsu, China). Briefly, colostrum samples were taken out, added with ultrapure water in a 1:4 volume ratio, homogenized, and centrifuged at 3,500 rpm for 10 min. The supernatant was taken for the detection of IgG content. Serum concentrations of LPS were determined using the Mouse LPS ELISA kit from Jiangsu Enzyme Tag Biotechnology Co., LTD (Jiangsu, China).

### Statistical analysis

Data are expressed as the mean ± SE (the standard error of the mean). Statistical analysis of the index was carried out according to the replicate of each group. A two-sided unpaired Student’s *t*-test with Benjamini–Hochberg correction was used to compare the two groups. The differences among the three groups were analyzed using one-way analysis of variance (ANOVA) followed by Duncan’s test. Significance was set at *p* < 0.05.

## Results

### Antibiotic treatment during pregnancy altered the composition of maternal gut microbiota

**Figure 1:**
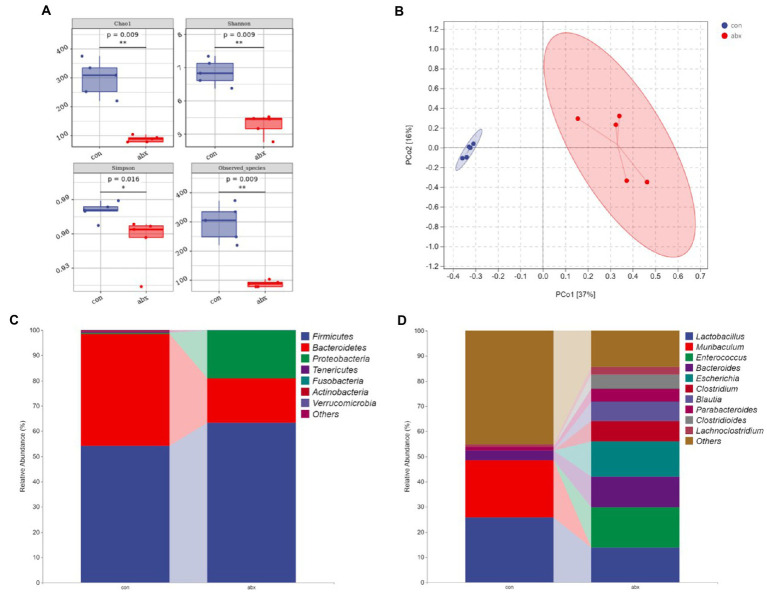
Effects of antibiotic treatment during pregnancy on gut microbiota. con: the Control group; abx: Group supplemented with antibiotics during pregnancy. **(A)** α diversity index; **(B)** PCoA clustering analysis; **(C,D)** Community taxonomic composition and abundance distribution map at the phylum and genus level.

Maternal gut microbiota can affect the production of maternal antibodies (Zeng et al., 2016; Zheng et al., 2020). In the present study, antibiotic treatment during pregnancy significantly reduced maternal cecal microbial richness (Chao1 and Obesrved species) and diversity (Shannon and Simpson; *p* < 0.05, Figure 1A). PCoA revealed a significant separation on the microbiota of the group of con and abx (Figure 1B). Moreover, the abx group sharply reduced the relative abundance of *Bacteroidetes*, *Proteobacteria*, and *Actinobacteria* (*p* < 0.05, Figure 1C and Supplementary Table S2), *reduced Firmicutes* but not significant. Additionally, the abx group remarkably decreased the relative abundance of *Muribaculum*, *increased Escherichia* (*p* < 0.05, Figure 1D and Supplementary Table S3), and had a tendency to increase *Enterococcus* and *Blautia* (*p* < 0.1, Figure 1D and Supplementary Table S3), as well as reduced *Lactobacillus*.

### Antibiotics treatment changed the bile acid levels of metabolites of maternal gut microbiota

**Figure 2:**
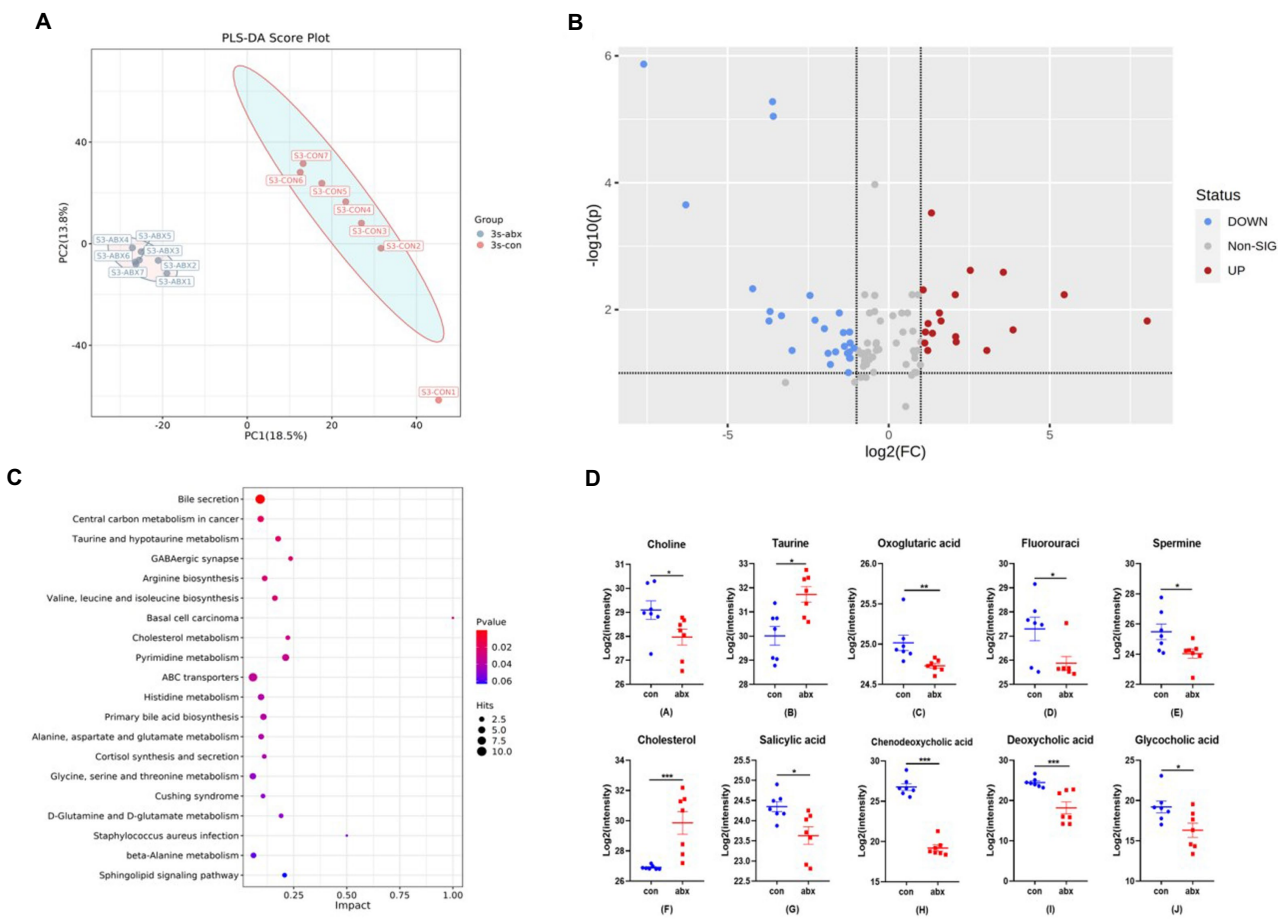
Effect of antibiotic treatment on plasma metabolites in maternal pregnancy. **(A)** PCA clustering analysis. **(B)** Volcano map. **(C)** Enrichment of KEGG metabolic pathway. **(D)** Differential metabolites in bile acid secretion pathway. Con: control group, abx: antibiotic treatment in maternal pregnancy.

Since gut microbiota was modulated by abx supplementation, we next investigated the effect of abx on microbial metabolites through plasma non-target metabolomics. PCA revealed a significant separation on the metabolites of the groups (Figure 2A). A total of 92 differential metabolites was found in the two groups, of which 18 were up-regulated and 26 were down-regulated (*p* < 0.05, Figure 2B). It can be seen from the metabolic pathway of KEGG enrichment that bile acid secretion is the most significant (Figure 2C). Abx treatment dramatically decreased the level of choline, oxoglutaric acid, fluorouraci, spermine, salicylic acid, chenodeoxycholic acid, deoxycholic acid and glycocholic acid, sharply increased the level of Taurine and cholesterol (*p* < 0.05, Figures 2C,D). Antibiotic treatment reduced the levels of primary bile acid glycylcholic acid, chenodeoxycholic acid, and secondary bile acid deoxycholic acid, and increased the level of cholesterol the raw material for bile acid synthesis (*p* < 0.05, Figure 2D).

### Antibiotic treatment affected maternal intestinal immunity

**Figure 3:**
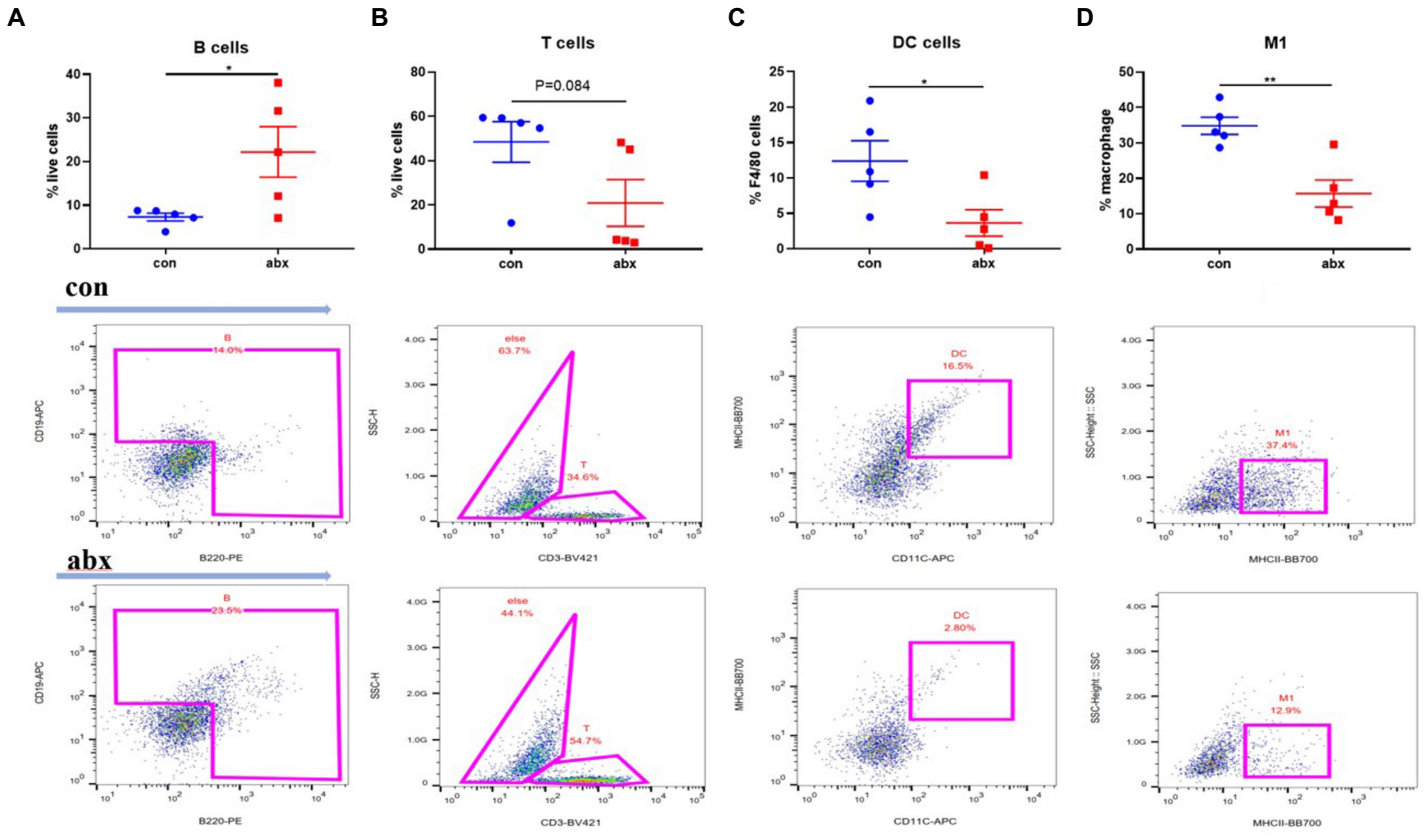
Effect of antibiotic treatment on maternal intestinal lamina propria immune cells. Antibiotic treatment significantly enhanced the number of B cells in intestinal lamina propria of the mother’s small intestine **(A)**, and reduced the number of T cells **(B)**, DC cells **(C)**, and M1 macrophages **(D)**.

Secondary bile acid was reported that affects intestinal immunity (Campbell et al., 2020; Song et al., 2020). Thus, the type and number of intestinal immune cells was detected. In the present study, antibiotic treatment during pregnancy could significantly increase the number of B cells (*p* < 0.05, Figure 3A) in the intestinal lamina propria of the mother, and reduce the number of T cells (Figure 3B) and DC cells (*p* < 0.05, Figure 3C), not affected the number of macrophages (*p* > 0.05, Supplementary Figure S1), but significantly reduces the number of M1 macrophages (*p* < 0.05, Figure 2D).

### Antibiotic treatment lowered the transport of maternal antibody IgG through FcRn receptor

**Figure 4:**
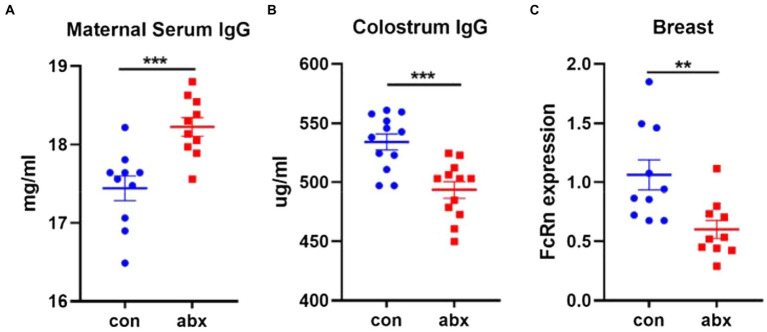
Antibiotic treatment affected the transmission of maternal antibody IgG. Antibiotic treatment significantly improved the level of maternal serum IgG **(A)**, lowered the level of Colostrum IgG **(B)**, and expression of FcRn in breast **(C)**.

Intestine is the largest immune organ of the body, and there are a large number of B cells (Kabat et al., 2014; Mowat and Agace, 2014) in the intestinal lamina propria. Therefore, the effect of antibiotic treatment on the production of maternal antibody IgG was evaluated. In the present study, antibiotic treatment remarkably improved the level of maternal serum IgG (*p* < 0.05, Figure 4A), while it surprisingly significantly reduced the level of IgG in colostrum (*p* < 0.05, Figure 4B). FcRn receptor plays an important role in the transport of IgG in maternal mammary gland and the absorption of IgG in neonatal small intestine (Zheng et al., 2020). Antibiotic treatment dramatically lowered the expression of FcRn, a specific IgG transporters (*p* < 0.05, Figure 4C).

### Toll like receptor 2/4 was an important target for IgG transport of maternal antibody IgG

**Figure 5:**
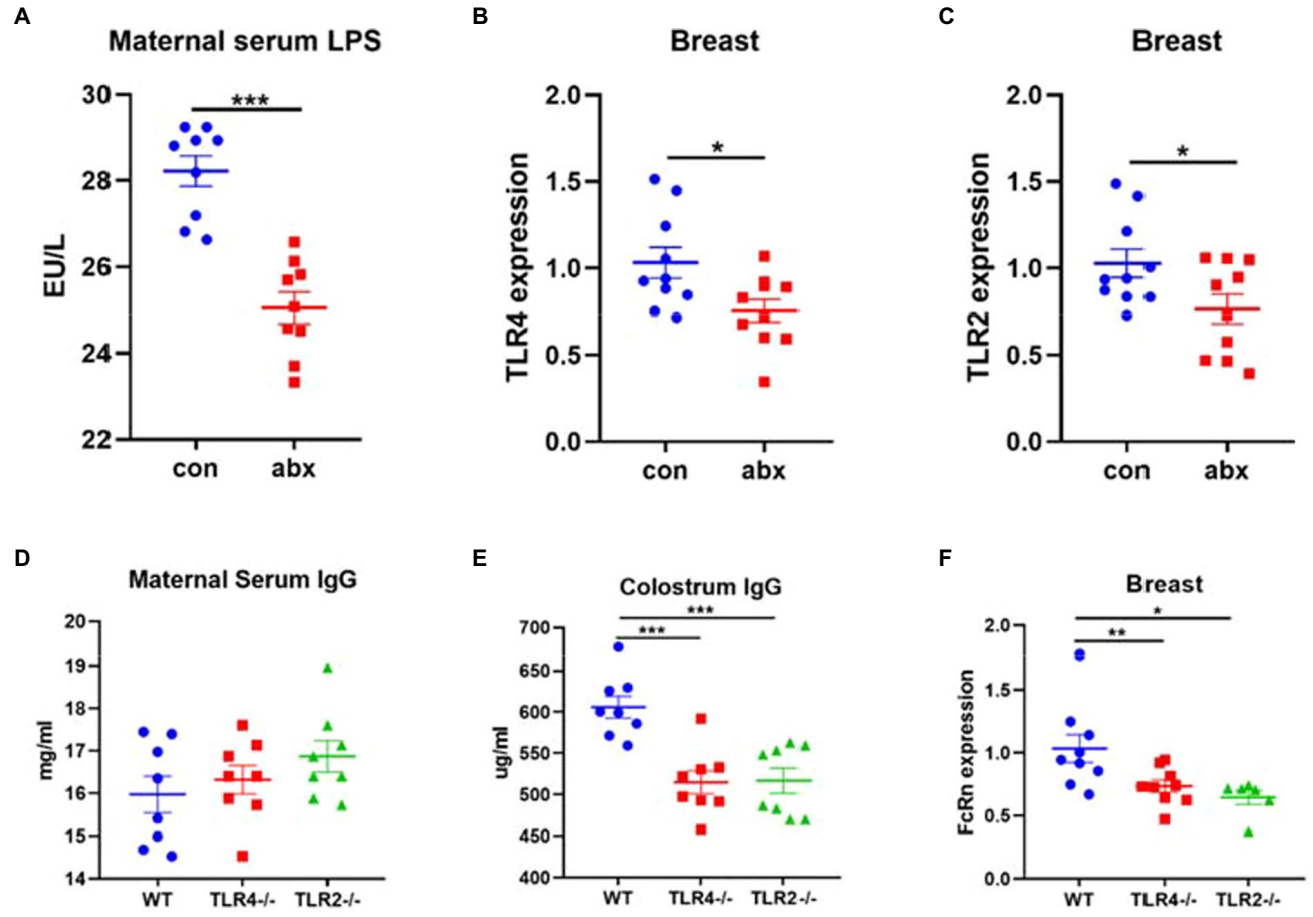
Antibiotic treatment affects the transmission of maternal antibody IgG through TLR2/4 receptor. **(A)** Maternal serum LPS. **(B,C)** TLR4 and TLR2 mRNA expression in breast. **(D-F)** Maternal serum IgG, colostrum IgG concentration and FcRn mRNA expression in breast of wild-type mice and TLR4 and TLR2 gene knockout mice.

NF-κB signaling pathway has been reported that plays an important role in the regulation of FcRn expression (Liu et al., 2007, 2008). As the upstream of NF-κB signal pathway, the effect of toll like receptor on the expression of FcRn has been rarely studied. In the present study, antibiotic treatment remarkably reduced the expression of TLR4 and TLR2 in breast (*p* < 0.05, Figures 5B,C). LPS is the main component of the cell wall of gram-negative bacteria and a TLR4 receptor agonist. Antibiotic treatment sharply reduced the level of maternal serum LPS (*p* < 0.05, Figure 5A). Moreover, compared with wild type mice, the serum IgG of TLR4 and TLR2 gene knockout mice did not change (Figure 5D), but significantly reduced the level of IgG in colostrum (*p* < 0.05, Figure 5E) and the mRNA expression of FcRn in breast (*p* < 0.05, Figure 5F).

### Antibiotic treatment affects the absorption of maternal antibodies in neonatal intestine by affecting the expression of TLR2 and TLR4

**Figure 6:**
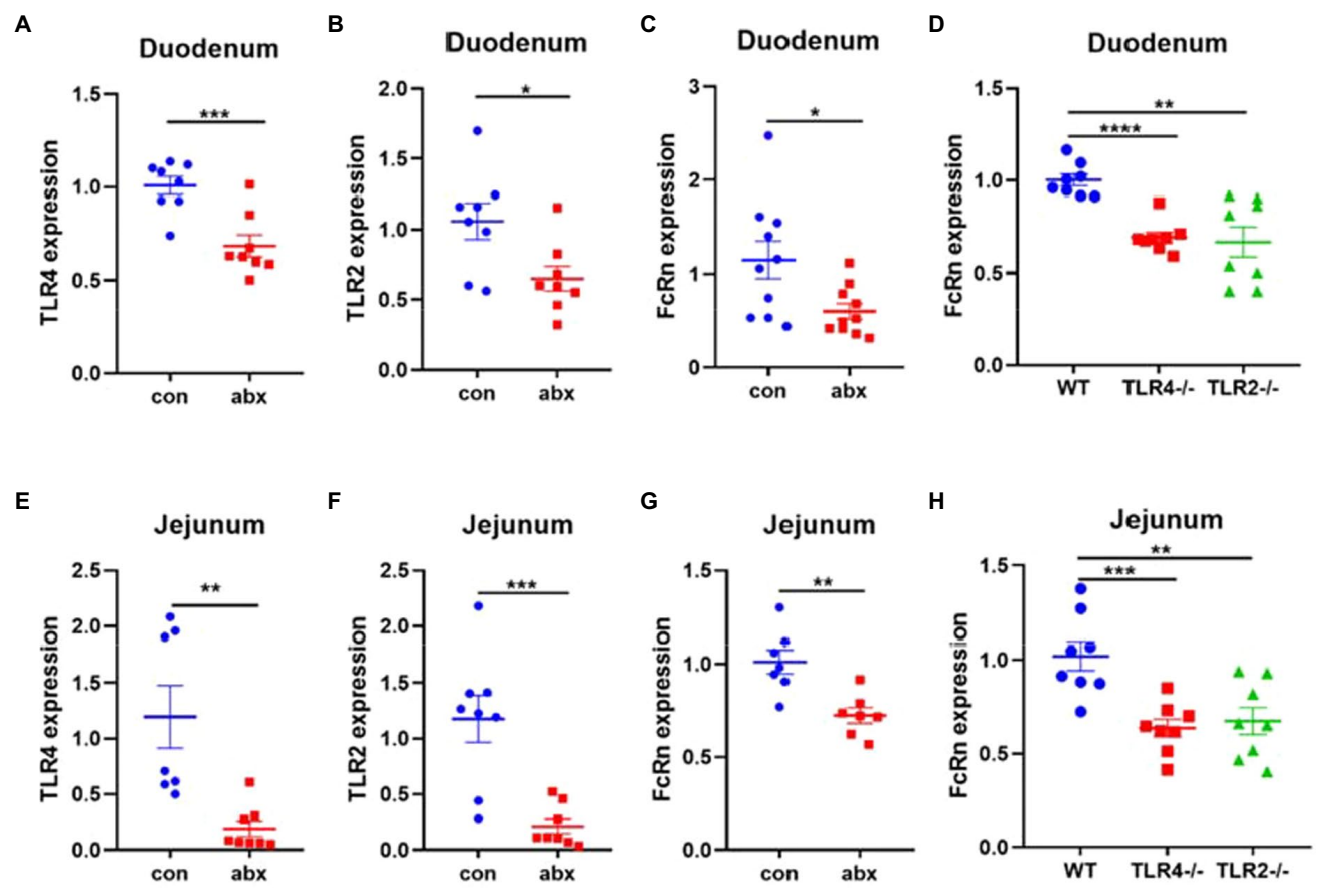
Antibiotic treatment affects the absorption of maternal antibodies IgG in pups. **(A–C)** TLR4, TLR2 and FcRn mRNA expression in duodenum of pups (3-day old). **(D)** FcRn mRNA expression in the duodenum of pups wild type and TLR4 and TLR2 knockout mice. **(E–G)** TLR4, TLR2 and FcRn mRNA expression in jejunum of pups. **(H)** FcRn mRNA expression in the jejunum of pups wild type and TLR4 and TLR2 knockout mice.

It is very important for neonates to get enough maternal antibodies from colostrum to resist the pathogen infection. In the present study, maternal antibiotic treatment significantly reduced the mRNA expression of TLR4, TLR2, and FcRn in duodenum and jejunum of pups (*p* < 0.05, Figures 6A–C,E–G). TLR4 and TLR2 knockout mice remarkably lowered FcRn mRNA expression in duodenum and jejunum of pups (*p* < 0.05, Figures 6D,H).

## Discussion

Among the numerous causes of death due to bacterial pathogens in children under 5 years old, acute infectious diarrhea is surpassed only by pneumonia (Kotloff et al., 2013). Neonatal diarrhea in developing countries is frequent, leading to high mortality, the major infectious agents, which accounts for around 1.5 million deaths annually, are ETEC, *rotavirus*, *Vibrio cholerae*, and *Shigella* (Kotloff et al., 2017, 2019). Epidemiological data demonstrated that breast-feeding lessen overall rates of diarrhea and mortality (Thapar and Sanderson, 2004; Qadri et al., 2005). Human and rodent milk contains substantial amounts of both secretory IgA and IgG, and the content of IgG in colostrum is higher (Goldsmith et al., 1983; Fouda et al., 2011). The breast cannot produce IgG, and the IgG in the milk is obtained by transferring serum IgG from the breast (Rezaei et al., 2016). Therefore, how to improve the level of serum IgG and how the breast transports serum IgG are very important.

The commensal microbiota can affect production of maternal antibodies (Macpherson et al., 2017; Chen et al., 2018). So, we treated with antibiotics during pregnancy to change the maternal gut microbiota. The results showed that antibiotic treatment during pregnancy significantly reduced maternal cecal microbial richness (Chao1 and Obesrved species) and diversity (Shannon and Simpson), and increased the level of serum IgG. IgG is mainly produced by B cells. Intestine is the largest immune organ of the body (Mowat and Agace, 2014). Intestinal lamina propria, as the place where B cells are abundant, plays an crucial role in the production of IgG (Kabat et al., 2014). Flow cytometry also found that antibiotic treatment increased the number of B cells in the intestinal lamina propria of dams, which was in line with the above-mentioned results. Generally, gut microbiota can regulate intestinal immune function *via* its metabolites. Therefore, the effect of abx on plasma metabolites was further investigated through plasma non-target metabolomics in our present study. Our research showed that bile acid secretion was the most significant from the metabolic pathway of KEGG enrichment, and reduced the levels of secondary bile acid deoxycholic acid in antibiotic treatment. Relevant studies also revealed that secondary bile acids, metabolites of gut microbiota, can affect intestinal immunity (Campbell et al., 2020; Song et al., 2020). These findings indicate that antibiotic treatment during pregnancy can affect the production of maternal antibody IgG, and affect the amount of intestinal lamina propria B cells by affecting gut microbiota and their metabolites.

It is surprisingly that antibiotic treatment increased the concentration of maternal serum IgG, the content of colostrum IgG was decreased. Colostrum IgG is transported by serum IgG through FcRn receptor of breast, a sole transporter (Lu et al., 2007; Bournazos and Ravetch, 2017). Therefore, we detected the expression of FcRn, an IgG transporter in the breast. The results showed that antibiotic treatment significantly downregulated the expression of FcRn, which confirmed that antibiotic treatment affected the transport of serum IgG by breast. In addition, LPS stimulated and NF-κB signaling pathway activation could upregulate the expression of FcRn (Liu et al., 2007, 2008). Whether LPS as agonist of TLR4 receptor and Toll like receptor as the upstream of NF-κB signaling pathway can affect the expression of FcRn has been rarely studied. The results of this study showed that antibiotic treatment significantly reduced the concentration of serum LPS and the mRNA expression of TLR4 and TLR2 in breast. The TLR4 and TLR2 gene knockout dams did not affect the content of serum IgG, but lowered the content of colostrum IgG and the mRNA expression of FcRn in breast. This indicates that TLR4 and TLR2 play an significant role in the process of IgG transport in the breast. Relevant studies have found that TLR4 and TLR2 gene knockout mice can reduce the serum IgG content of mice under steady-state conditions, which is inconsistent with the results of this study (Zeng et al., 2016). The reason may be that the mice used in this experiment are pregnant mice, and their gut microbiota are in the process of dynamic change (Gagnebin et al., 2017).

Newborns can transfer IgG in colostrum to serum through intestinal FcRn receptor, and obtain immune protection (Zheng et al., 2020). About 80% of the IgG absorbed in the small intestine is mediated by FcRn protein expressed on the surface of small intestinal chorion, while the remaining IgG is absorbed through non-specific pinocytosis (Kliwinski et al., 2013). In this study, antibiotic treatment remarkably decreased the expression of FcRn mRNA in the duodenum and jejunum of pups, and correspondingly decreased the expression of TLR4 and TLR2 mRNA. In addition, the offspring of TLR4 and TLR2 knockout dams also found that the intestinal FcRn expression was downregulated. This result indicated that TLR4 and TLR2 also plays an important role in the absorption of intestinal IgG in neonates.

## Conclusion

Antibiotic treatment during pregnancy changed the composition of cecum microorganisms (reduced Chao1, Observed species, Shannon, Simpson indexes), decreased the concentration of secondary bile acid, a metabolite of microorganisms, and increased the number of intestinal lamina propria B cells and the concentration of serum IgG. Moreover, antibiotic treatment during pregnancy downregulated the expression of FcRn receptor in breast, that is, lowered the transport of serum IgG to colostrum, and diluted the concentration of colostrum IgG. It is also confirmed that TLR4 and TLR2 play an important role in the transport and absorption of IgG.

## Data availability statement

The datasets presented in this study can be found in online repositories. The names of the repository/repositories and accession number(s) can be found below: NCBI SRA, accession number: PRJNA925114.

## Ethics statement

The animal study was reviewed and approved by The animal study was reviewed and approved by Ethics of Animal Experiments of Hunan Agriculture University.

## Author contributions

YD, XY, and XH: conceptualization. YD: methodology, investigation all experiments, and writing—original draft. HZ, XH, and ZS: mass-spectrum assays. ZS: flow cytometric analysis. HZ, XY, and ZS: formal analysis. ZS: funding acquisition and resources. XH and HZ: supervision. All authors contributed to the article and approved the submitted version.

## Funding

The authors are thankful to the Project supported by the National Natural ScienceFoundation of China (Grant No: 31902171).

## Conflict of interest

The authors declare that the research was conducted in the absence of any commercial or financial relationships that could be construed as a potential conflict of interest.

## Publisher’s note

All claims expressed in this article are solely those of the authors and do not necessarily represent those of their affiliated organizations, or those of the publisher, the editors and the reviewers. Any product that may be evaluated in this article, or claim that may be made by its manufacturer, is not guaranteed or endorsed by the publisher.

## Supplementary material

The Supplementary material for this article can be found online at: https://www.frontiersin.org/articles/10.3389/fmicb.2023.1109273/full#supplementary-material

## Footnotes

^1^ http://www.hmdb.ca

^2^ http://www.massbank.jp/

^3^ http://www.lipidmaps.org

^4^ https://www.mzcloud.org

^5^ http://www.genome.jp/kegg/
