# Supplementary material for: Maternal antibiotic treatment during pregnancy attenuates the transport and absorption of maternal antibody IgG through TLR4 and TLR2 receptor
Source: Front Microbiol. 2023 Feb 17;14:1109273. doi: 10.3389/fmicb.2023.1109273 (PMC9986424; doi:10.3389/fmicb.2023.1109273)
Supplement: Supplementary file 1 [file Data_Sheet_1.docx]

**Supplementary figures and tables**

Table S1 Table 2 Primer sequences used for real-time quantitative PCR.

| Genes^1^ | Primer sequence (5’-3’) |
| --- | --- |
| GADPH F | AGGTCGGTGTGAACGGATTTG |
| GADPH R | TGTAGACCATGTAGTTGAGGTCA |
| TLR4 F | TCTGGGGAGGCACATCTTCT |
| TLR4 R | AGGTCCAAGTTGCCGTTTCT |
| TLR2 F | AGCCCATTGAGAGGAAAGCC |
| TLR2 R | CCAAAACACTTCCTGCTGGC |
| FcRn F | AAATGGTCAGAAGAGGGGGAC |
| FcRn R | CCTCACCATTGAGGGCAAAC |

^1^ F, Forward primer; R, Reverse primer*.*

Table S2 Effect of antibiotic treatment on the composition of maternal gut microbiota (phylum level)

| Item | con | abx | p Value |
| --- | --- | --- | --- |
| *Firmicutes* | 0.54±0.071 | 0.63±0.083 | 0.427 |
| *Bacteroidetes* | 0.44±0.070 | 0.17±0.076 | 0.031 |
| *Proteobacteria* | 0.0037±0.0010 | 0.19±0.038 | 0.001 |
| *Tenericutes* | 0.0033±0.0020 | 0.000±0.000 | 0.147 |
| *Fusobacteria* | 0.000±0.000 | 0.0022±0.0016 | 0.192 |
| *Actinobacteria* | 0.0020±0.00044 | 0.000±0.000 | 0.002 |

con: Control group. abx: Group supplemented with antibiotics during pregnancy. Data expressed as mean ± standard error.

Table S3 Effect of antibiotic treatment on the composition of maternal gut microbiota (species level)

| Item | con | abx | p Value |
| --- | --- | --- | --- |
| *Lactobacillus* | 0.258±0.050 | 0.138±0.048 | 0.122 |
| *Muribaculum* | 0.228±0.040 | 0.0002±0.00015 | 0.005 |
| *Enterococcus* | 0.000±0.000 | 0.16±0.060 | 0.057 |
| *Bacteroides* | 0.0372±0.015 | 0.121±0.075 | 0.329 |
| *Escherichia* | 0.0003±0.00018 | 0.140±0.039 | 0.022 |
| *Clostridium* | 0.0001±0.00009 | 0.0817±0.043 | 0.131 |
| *Blautia* | 0.00001±0.00008 | 0.0764±0.035 | 0.095 |
| *Parabacteroides* | 0.0142±0.0073 | 0.0525±0.052 | 0.506 |
| *Clostridioides* | 0.000±0.000 | 0.0566±0.052 | 0.340 |
| *Lachnoclostridium* | 0.0107±0.0033 | 0.0288±0.011 | 0.171 |

**con:** Control group; **abx:** Group supplemented with antibiotics during pregnancy. Data expressed as mean ± standard error.


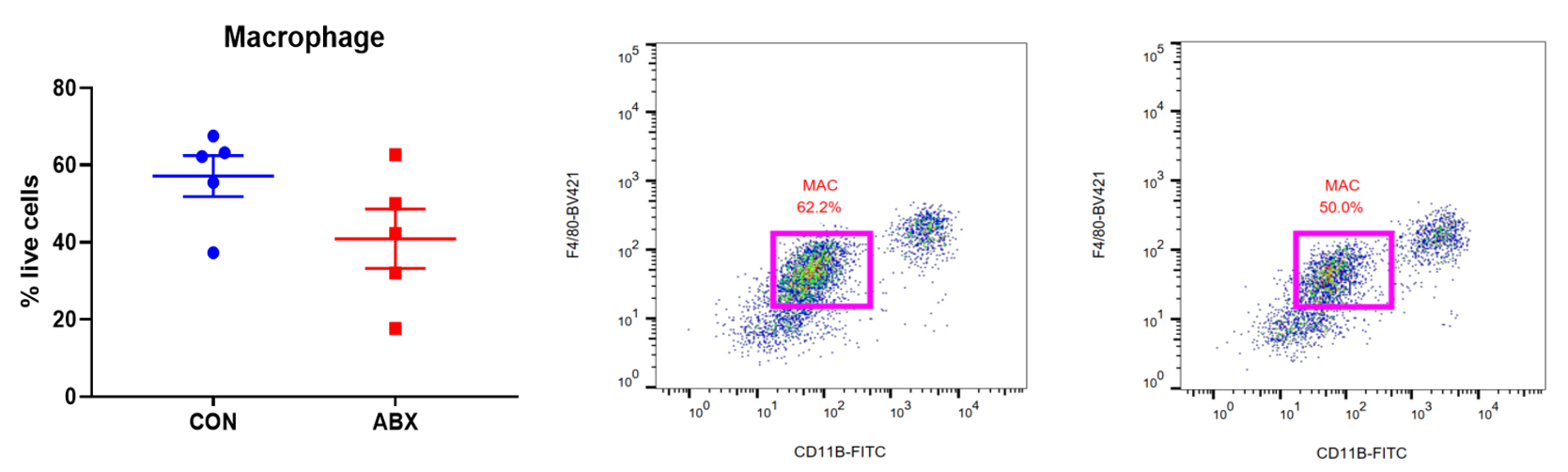


FIGURE S1 Effect of antibiotic treatment on maternal intestinal lamina propria macrophage cells.
